# Complications and Costs Associated With Ethnicity Following Total Hip Arthroplasty: A Retrospective Matched Cohort Study

**DOI:** 10.7759/cureus.40595

**Published:** 2023-06-18

**Authors:** Vikram A Aggarwal, Garrett Sohn, Sharon Walton, Senthil Sambandam, Dane Wukich

**Affiliations:** 1 Orthopedics, University of Texas Southwestern Medical Center, Dallas, USA

**Keywords:** retrospective cohort, race inequities, healthcare costs, complications, total hip athroplasty

## Abstract

Background: Minority patients often have greater numbers of complications, revisions, and costs after total hip arthroplasty (THA). This study investigates how race correlates with specific surgical complications, revisions, and total costs following THA both before and after propensity matching.

Methods: Data from 2014-2016 were collected from a large commercial insurance database known as PearlDiver. THA patients were assigned under Current Procedural Terminology (CPT-27130) and International Statistical Classification of Diseases (ICD-9-P-8151) codes and then divided into groups based on racial status in the database. Patients of different ethnicities including White, Black, Asian, and Hispanic patients were compared in regard to age, gender, comorbidities, lengths of stay, and surgical complications and costs at thirty days, ninety days, and one year using unequal variance t-tests. Black, Asian, and Hispanic patients are collectively referred to as minority patients. Patient comparisons were done both before and after matching for age, gender, tobacco use, diabetes, and obesity comorbidities.

Results: A total of 73,688 White (93%), 4,822 Black (6%), 268 Asian (0.3%), and 420 Hispanic (0.5%) THA patients were included. Significantly more minority patients underwent THA under the age of 65 and had higher comorbidity indices and lengths of stay. Black patients had significantly higher complication rates, but there was no significant difference in rates of revision in any minority group. Minority patients were charged 9%-83% more. After matching, Black and Hispanic patients maintained higher comorbidity indices and lengths of stay. Black patients had a spectrum of complication rates but significantly decreased revision rates. Furthermore, after matching, minority patients were charged 5%-65% more.

Conclusions: Black patients experienced significantly greater rates of complications and higher total costs; whereas, Asian and Hispanic patients did not have significant differences in complications but did have higher costs. Therefore, this study aligns with previous studies and supports our hypothesis that Black ethnicity patients have worse outcomes than White ethnicity patients after THA, advocating for reducing health disparities and establishing more equitable healthcare, but does not support our hypothesis for Asian and Hispanic patients, likely due to a small study population size, warranting further research into the topic.

## Introduction

Total hip replacement surgery has become one of the most common orthopaedic surgeries done today and is projected to increase by 71% to 635,000 primary hip arthroplasties by 2030 [1]. Furthermore, patients report substantial functional improvement and pain reduction following hip replacement [2]. Even so, while minority patients have a higher prevalence of arthritis-related-activity-limitation [3], Black, Asian, and Hispanic patients are less likely to utilize hip replacement surgery, including revision arthroplasty, than their White counterparts with various explanations given, ranging from differences in health literacy, socioeconomic status (SES), and increased post-operative complication rates [4-7]. Previous studies have supported this phenomenon of minority patients having higher complication rates than their White counterparts [8-10]. These potentially increased complications and revisions can lead to greater costs of care following hip replacement, and studies by Delanois et al. [11] and Chisari et al. [12] found that minority patients had higher costs of care at thirty days and ninety days, respectively, however, few studies report on cost at one year.

To the best of our knowledge, there are no prior studies examining post-operative complication rates along with total costs of hip replacement between various racial groups with and without propensity matching. Therefore, we aimed to investigate differences in post-operative complications and total costs following total hip arthroplasty (THA) between Black, Asian, and Hispanic patients compared to White patients before and after matching at thirty days, ninety days, and one year. We hypothesize that Black, Asian, and Hispanic patients, also referred to as minority patients, will have higher rates of complications and costs than their White counterparts.

## Materials and methods

Data source and study population

This retrospective study queried the Medicare Standard Analytic Files of the PearlDiver Patient Records Database (www.pearldiverinc.com, Colorado Springs, CO, USA) for all patients who underwent a hip replacement using Current Procedural Terminology (CPT-27130) and International Statistical Classification of Diseases (ICD-9-P-8151) codes between 2014 and 2016. The Medicare Standard Analytic Files of the PearlDiver database contains longitudinal records for 51 million covered lives with both inpatient and outpatient information on charges and reimbursements. Patient information from all sources was de-identified and compliant with the Health Insurance Portability and Affordability Act (HIPAA). Therefore, the study did not require review by our institutional review board (IRB).

We included patients who underwent primary hip replacement during the period specified above, and who also maintained at least one year of post-operative follow-up in the PearlDiver database. Patients with less than one year of postoperative follow-up were excluded from the study. Patients were then further divided based on whether they were self-reported as being White, Black, Asian, or Hispanic.

Patient characteristics

White, Black, Asian, and Hispanic patients were initially compared at baseline before any matching was done. This comparison included the Charlson Comorbidity Index (CCI), Elixhauser Comorbidity Index (ECI), and length of stay (LOS). Subsequently, Bellwether-PearlDiver was used to generate propensity-matched populations from each Black, Asian, or Hispanic population and White population based on age, gender, tobacco use, diabetes, and obesity (defined as body mass index (BMI) greater than 30 kg/m^2^) comorbidities. This resulted in three separate minority patient cohorts with a corresponding White patient cohort for each with an equal number of patients in each and similar age, gender, and proportions of tobacco use, diabetes, and obesity comorbidities.

Post-operative complications

The following post-operative complications were assessed between the groups over the time spans of thirty days, ninety days, and one year: dislocation, surgical site infection (SSI), deep venous thrombosis (DVT), wound disruption, hematoma, pulmonary embolism (PE), blood transfusion, and readmission. Revisions were assessed at two years post-operative time. These complications were chosen a priori. The odds ratio of complications for each group was calculated and compared to the corresponding White group standard, along with 95% confidence intervals (CI), and the p value for each complication. Values less than 11 were outputted as “-1” from the PearlDiver system. Therefore, for any complications with -1 as a value, the odds ratio and p value could not be calculated, and those complications have been listed as 'NA'.

Total cost of care

The total costs of care were calculated over the time spans of thirty days, ninety days, and one year. The means and standard deviations (SD) of the total cost of all groups were then calculated and compared to the corresponding White group standard, along with the 95% CI and p value for each span of time.

Statistical methods

Patient characteristics were described using mean (avg) and SD for continuous variables and frequency (n) and proportion (%) for categorical variables where appropriate. Two-independent sample t-test for unequal variances (continuous variables) and a test of significance was used to identify any differences between the two groups. Statistical analyses were performed using MedCalc’s Odds Ratio Calculator and MedCalc’s Comparison of Means Calculator [13,14]. Statistical significance was defined as a p value < 0.05.

## Results

Unmatched cohort

A total of 1,254,696 patients underwent THA during the selected time frame. Of those, 113,987 were listed as White, 7,615 as Black, 479 as Asian, and 671 as Hispanic. From there, 73,688 White (93%), 4,822 Black (6.09%), 268 Asian (0.34%), and 420 Hispanic (0.53%) patients who had at least a year of follow-up were included under hip replacement. Minority patients were 2-7.6 times more likely to be under 65 years old and had significantly increased CCI, ECI, and LOS values and higher rates of diabetes. Asian patients had significantly lower rates of obesity and both Asian and Hispanic patients had lower rates of tobacco use. There was a similar gender distribution between White, Black, and Hispanic patients, but there were more female than male Asian patients (Table 1).

**Table 1:** Patient Demographics CCI: Charlson Comorbidity Index; ECI: Elixhauser Comorbidity Index; LOS: length of stay

	White	Black	Comparison	CI	P Value	Asian	Comparison	CI	P Value	Hispanic	Comparison	CI	P Value
Total n (%)	73688 (93.0)	4822 (6.09)				268 (0.34)				420 (0.53)			
Under 65 n (%)	6606 (9.00)	1747 (36.2)	5.769	5.412 - 6.15	< 0.0001	44 (16.4)	1.995	1.442 - 2.759	< 0.0001	180 (42.9)	7.616	6.267 - 9.255	< 0.0001
Male n (%)	27102 (36.8)	1742 (36.1)	0.972	0.915 - 1.033	0.362	69 (25.7)	0.596	0.453 - 0.784	0.0002	161 (38.3)	1.069	0.877 - 1.302	0.5103
CCI avg (SD)	1.82 (2.19)	2.52 (2.69)	1.385	1.349 - 1.42	< 0.0001	2.00 (2.47)	1.099	0.955 - 1.243	0.1795	2.55 (2.86)	1.401	1.285 - 1.517	< 0.0001
ECI avg (SD)	5.25 (3.57)	6.25 (4.02)	1.19	1.17 - 1.21	< 0.0001	5.05 (3.61)	0.962	0.880 - 1.043	0.36	6.26 (4.14)	1.192	1.127 - 1.258	< 0.0001
LOS avg (SD)	2.83 (1.71)	3.15 (2.30)	1.112	1.094 - 1.13	< 0.0001	3.28 (2.30)	1.158	1.088 - 1.230	< 0.0001	3.36 (1.90)	1.184	1.126 - 1.242	< 0.0001
Obesity n (%)	25753 (34.9)	2319 (48.1)	1.725	1.627 - 1.828	< 0.0001	50 (18.7)	0.427	0.314 - 0.581	< 0.0001	182 (43.3)	1.423	1.173 - 1.727	0.0004
Diabetes n (%)	30663 (41.6)	2636 (54.7)	1.692	1.596 - 1.794	< 0.0001	147 (54.9)	1.705	1.34 - 2.169	< 0.0001	244 (58.1)	1.945	1.602 - 2.363	< 0.0001
Tobacco Use n (%)	36779 (49.9)	2790 (57.9)	1.378	1.299 - 1.462	< 0.0001	83 (31.0)	0.45	0.347 - 0.584	< 0.0001	165 (39.3)	0.649	0.534 - 0.790	< 0.0001

At both thirty and ninety days following THA, Black patients had significantly higher rates of SSI, hematoma, transfusion, and readmission. One year following arthroplasty, Black patients had significantly higher rates of DVT and PE, along with previously observed increases in hematoma, transfusion, and readmission. At one year, Hispanic patients had significantly higher rates of transfusion (Tables 2-4).

**Table 2:** Unmatched Post-Operative Complications 30 Days after Total Hip Arthroplasty (THA) SSI: surgical site infection; DVT: deep venous thrombosis; PE: pulmonary embolism

	White	Black	Odds Ratio	CI	P Value	Asian	Odds Ratio	CI	P Value	Hispanic	Odds Ratio	CI	P Value
Dislocation n (%)	722 (0.98)	54 (1.12)	1.145	0.867 - 1.512	0.3412	-1 (NA)	NA	NA	NA	-1 (NA)	NA	NA	NA
SSI n (%)	1059 (1.44)	90 (1.87)	1.304	1.05 - 1.621	0.0165	-1 (NA)	NA	NA	NA	-1 (NA)	NA	NA	NA
DVT n (%)	57 (0.08)	-1 (NA)	NA	NA	NA	-1 (NA)	NA	NA	NA	-1 (NA)	NA	NA	NA
Wound Disruption n (%)	302 (0.41)	27 (0.56)	1.368	0.922 - 2.031	0.1195	-1 (NA)	NA	NA	NA	-1 (NA)	NA	NA	NA
Hematoma n (%)	665 (0.90)	61 (1.27)	1.407	1.081 - 1.832	0.0112	-1 (NA)	NA	NA	NA	-1 (NA)	NA	NA	NA
PE n (%)	332 (0.45)	20 (0.41)	0.920	0.586 - 1.446	0.719	-1 (NA)	NA	NA	NA	-1 (NA)	NA	NA	NA
Transfusion n (%)	825 (1.12)	91 (1.89)	1.699	1.365 - 2.114	< 0.0001	-1 (NA)	NA	NA	NA	-1 (NA)	NA	NA	NA
Readmission n (%)	3182 (4.33)	319 (6.63)	1.571	1.395 – 1.770	< 0.0001	15 (5.59)	1.311	0.778 – 2.210	0.3087	26 (6.19)	1.460	0.980 – 2.174	0.0628

**Table 3:** Unmatched Post-Operative Complications 90 Days after Total Hip Arthroplasty (THA) SSI: surgical site infection; DVT: deep venous thrombosis; PE: pulmonary embolism

	White	Black	Odds Ratio	CI	P Value	Asian	Odds Ratio	CI	P Value	Hispanic	Odds Ratio	CI	P Value
Dislocation n (%)	1239 (1.7)	77 (1.6)	0.949	0.752 - 1.197	0.6577	-1 (NA)	NA	NA	NA	-1 (NA)	NA	NA	NA
SSI n (%)	1602 (2.2)	127 (2.6)	1.217	1.014 - 1.462	0.0354	-1 (NA)	NA	NA	NA	-1 (NA)	NA	NA	NA
DVT n (%)	169 (0.23)	12 (0.25)	1.085	0.604 - 1.951	0.7844	-1 (NA)	NA	NA	NA	-1 (NA)	NA	NA	NA
Wound Disruption n (%)	518 (0.70)	42 (0.87)	1.241	0.905 - 1.702	0.18	-1 (NA)	NA	NA	NA	-1 (NA)	NA	NA	NA
Hematoma n (%)	874 (1.2)	81 (1.7)	1.423	1.131 - 1.791	0.0026	-1 (NA)	NA	NA	NA	-1 (NA)	NA	NA	NA
PE n (%)	539 (0.73)	43 (0.89)	1.221	0.894 - 1.668	0.2095	-1 (NA)	NA	NA	NA	-1 (NA)	NA	NA	NA
Transfusion n (%)	1299 (1.7)	149 (3.1)	1.777	1.496 - 2.111	< 0.0001	-1 (NA)	NA	NA	NA	11 (2.6)	1.499	0.821 - 2.735	0.1872
Readmission n (%)	6713 (9.13)	565 (11.7)	1.326	1.210 – 1.452	< 0.0001	22 (8.21)	0.891	0.575 – 1.3786	0.6033	45 (10.7)	1.195	0.876 – 1.630	0.2603

**Table 4:** Unmatched Post-Operative Complications One Year after Total Hip Arthroplasty (THA) SSI: surgical site infection; DVT: deep venous thrombosis; PE: pulmonary embolism

	White	Black	Odds Ratio	CI	P Value	Asian	Odds Ratio	CI	P Value	Hispanic	Odds Ratio	CI	P Value
Dislocation n (%)	1677 (2.3)	103 (2.1)	0.937	0.767 - 1.146	0.5277	-1 (NA)	NA	NA	NA	-1 (NA)	NA	NA	NA
SSI n (%)	2481 (3.4)	184 (3.8)	1.139	0.977 - 1.326	0.0956	-1 (NA)	NA	NA	NA	16 (3.81)	1.137	0.689 - 1.876	0.6164
DVT n (%)	723 (0.98)	70 (1.5)	1.487	1.161 - 1.903	0.0017	-1 (NA)	NA	NA	NA	-1 (NA)	NA	NA	NA
Wound Disruption n (%)	759 (1.0)	60 (1.2)	1.211	0.929 - 1.577	0.1566	-1 (NA)	NA	NA	NA	-1 (NA)	NA	NA	NA
Hematoma n (%)	1264 (1.7)	109 (2.3)	1.325	1.087 - 1.615	0.0053	-1 (NA)	NA	NA	NA	-1 (NA)	NA	NA	NA
PE n (%)	674 (0.92)	58 (1.2)	1.319	1.007 - 1.727	0.0444	-1 (NA)	NA	NA	NA	-1 (NA)	NA	NA	NA
Transfusion n (%)	2378 (3.2)	297 (6.2)	1.968	1.738 - 2.229	< 0.0001	-1 (NA)	NA	NA	NA	22 (5.24)	1.658	1.077 - 2.551	0.0216
Readmission n (%)	19,514 (26.5)	1543 (32.1)	1.3082	1.229 – 1.393	< 0.0001	67 (25.0)	0.923	0.700 – 1.218	0.5723	126 (30.0)	1.187	0.963 – 1.464	0.1083

Furthermore, minority patients were not more likely to require hip revision (Table 5).

**Table 5:** Unmatched Total Hip Arthroplasty (THA) Revision Rates at Two Years

	White	Black	Odds Ratio	CI	P Value	Asian	Odds Ratio	CI	P Value	Hispanic	Odds Ratio	CI	P Value
Revisions n (%)	1610 (2.2)	105 (2.2)	1.000	0.816 – 1.217	0.9729	-1 (NA)	NA	NA	NA	-1 (NA)	NA	NA	NA

All minority patients had significantly higher costs that became relatively higher across each time point, going from 9% at thirty days for Black patients to as high as 83% at one year for Hispanic patients (Table 6).

**Table 6:** Unmatched Total Costs after Total Hip Arthroplasty (in USD)

	White	Black	Comparison	CI	P Value	Asian	Comparison	CI	P Value	Hispanic	Comparison	CI	P Value
30 days avg (SD)	67169.50 (+/-46028.89)	73311.20 (+/-57056.94)	1.091	1.071 - 1.112	< 0.0001	93974.40 (+/- 68114.61)	1.400	1.317 - 1.481	< 0.0001	96463.89 (+/-67195.27)	1.436	1.370 - 1.502	< 0.0001
90 days avg (SD)	74310.86 (+/-57412.93)	85741.72 (+/-81691.62)	1.154	1.131 - 1.177	< 0.0001	104787.58 (+/- 87121.02)	1.410	1.317 -1.503	< 0.0001	111558.13 (+/-97736.22)	1.501	1.427 - 1.576	< 0.0001
1 year avg (SD)	113792.5 (+/-133987.20)	161899.74 (+/-277813.50)	1.423	1.385 - 1.460	< 0.0001	172509.12 (+/-276239.60)	1.516	1.374 - 1.658	< 0.0001	208616.11 (+/-371026.20)	1.833	1.718 - 1.948	< 0.0001

Matched cohort

A total of 4,822 Black and 4,822 White patients, 268 Asian and 268 White patients, and 420 Hispanic and 420 White patients were included under hip replacement after matching as described in the methods section. Age, gender, and rates of obesity, diabetes, and tobacco use were equalized. Despite this matching, CCI, ECI, and LOS were still significantly increased in Black and Hispanic patients, but not significantly in Asian patients (Table 7).

**Table 7:** Matched Patient Demographics CCI: Charlson Comorbidity Index; ECI: Elixhauser Comorbidity Index; LOS: length of stay

	White	Black	Comparison	CI	P Value	White	Asian	Comparison	CI	P Value	White	Hispanic	Comparison	CI	P Value
Total n (%)	4822 (43.8)	4822 (43.8)				268 (2.43)	268 (2.43)				420 (3.81)	420 (3.81)			
Under 65 n (%)	1742 (36.1)	1742 (36.1)	1.000	0.920 – 1.087	1.0000	44 (16.4)	44 (16.4)	1.000	0.633 – 1.579	1.0000	180 (42.9)	180 (42.9)	1.000	0.761 – 1.314	1.0000
Male n (%)	1747 (36.2)	1747 (36.2)	1.000	0.920 – 1.087	1.0000	69 (25.7)	69 (25.7)	1.000	0.679 – 1.473	1.0000	161 (38.3)	161 (38.3)	1.000	0.757 – 1.321	1.0000
CCI avg (SD)	2.11 (2.38)	2.52 (2.69)	1.194	1.146 - 1.242	< 0.0001	1.80 (2.20)	2.00 (2.47)	1.111	0.891 - 1.332	0.3227	1.91 (2.25)	2.55 (2.86)	1.335	1.152 - 1.518	0.003
ECI avg (SD)	5.85 (3.86)	6.25 (4.02)	1.068	1.041 - 1.095	< 0.0001	5.42 (3.78)	5.05 (3.61)	0.932	0.816 - 1.047	0.247	5.74 (3.55)	6.26 (4.14)	1.091	1.000 - 1.182	0.051
LOS avg (SD)	2.91 (1.74)	3.15 (2.30)	1.085	1.057 - 1.113	< 0.0001	3.11 (1.96)	3.28 (2.30)	1.057	0.940 - 1.173	0.3399	2.98 (1.49)	3.36 (1.90)	1.126	1.487 - 1.204	0.0015
Obesity n (%)	2319 (48.1)	2319 (48.1)	1.000	0.923 – 1.083	1.0000	50 (18.7)	50 (18.7)	1.000	0.648 – 1.544	1.0000	182 (43.3)	182 (43.3)	1.000	0.761 – 1.314	1.0000
Diabetes n (%)	2636 (54.7)	2636 (54.7)	1.000	0.923 – 1.084	1.0000	147 (54.9)	147 (54.9)	1.000	0.712 – 1.405	1.0000	244 (58.1)	244 (58.1)	1.000	0.760 – 1.315	1.0000
Tobacco Use n (%)	2790 (57.9)	2790 (57.9)	1.000	0.922 – 1.084	1.0000	83 (31.0)	83 (31.0)	1.000	0.693 – 1.442	1.0000	165 (39.3)	165 (39.3)	1.000	0.758 – 1.319	1.0000

**Figure 1:**
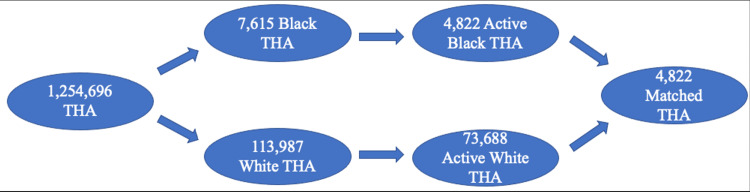
Patient Collection Schematic for Comparing White and Black Patients THA: total hip arthroplasty

**Figure 2:**
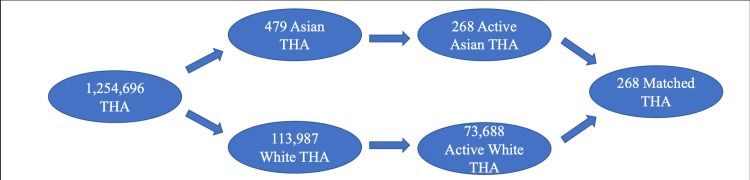
Patient Collection Schematic for Comparing White and Asian Patients THA: total hip arthroplasty

**Figure 3:**
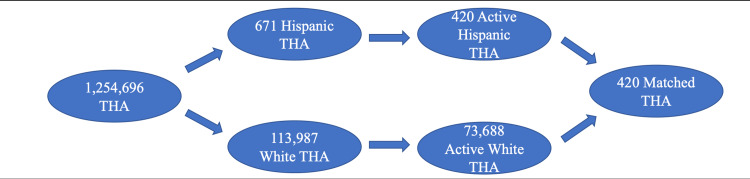
Patient Collection Schematic for Comparing White and Hispanic Patients THA: total hip arthroplasty

The process of collecting patients and creating comparison groups are summarized in Figures 1-3.

At ninety days, Black patients had a significantly higher rate of transfusion. At one year, Black patients had significantly higher rates of transfusion, but lower rates of dislocation and SSI (Tables 8-10).

**Table 8:** Matched Post-Operative Complications 30 Days after Total Hip Arthroplasty (THA) SSI: surgical site infection; DVT: deep venous thrombosis; PE: pulmonary embolism

	White	Black	Comparison	CI	P Value	White	Asian	Comparison	CI	P Value	White	Hispanic	Comparison	CI	P Value
Dislocation n (%)	61 (1.27)	54 (1.12)	0.884	0.612 - 1.278	0.5116	-1 (NA)	-1 (NA)	NA	NA	NA	-1 (NA)	-1 (NA)	NA	NA	NA
SSI n (%)	95 (1.97)	90 (1.87)	0.946	0.707 - 1.266	0.7105	-1 (NA)	-1 (NA)	NA	NA	NA	11 (2.62)	-1 (NA)	NA	NA	NA
DVT n (%)	-1 (NA)	-1 (NA)	NA	NA	NA	-1 (NA)	-1 (NA)	NA	NA	NA	-1 (NA)	-1 (NA)	NA	NA	NA
Wound Disruption n (%)	34 (0.71)	27 (0.56)	0.793	0.478 - 1.316	0.3696	-1 (NA)	-1 (NA)	NA	NA	NA	-1 (NA)	-1 (NA)	NA	NA	NA
Hematoma n (%)	60 (1.24)	61 (1.27)	1.017	0.710 - 1.456	0.9271	-1 (NA)	-1 (NA)	NA	NA	NA	-1 (NA)	-1 (NA)	NA	NA	NA
PE n (%)	31 (0.64)	20 (0.41)	0.644	0.366 - 1.131	0.1255	-1 (NA)	-1 (NA)	NA	NA	NA	-1 (NA)	-1 (NA)	NA	NA	NA
Transfusion n (%)	72 (2.63)	91 (1.89)	1.269	0.929 - 1.733	0.1342	-1 (NA)	-1 (NA)	NA	NA	NA	-1 (NA)	-1 (NA)	NA	NA	NA
Readmission n (%)	271 (5.67)	317 (6.64)	1.812	1.000 – 1.397	0.0504	19 (7.09)	15 (5.60)	0.777	0.386 – 1.563	0.4794	27 (6.43)	26 (6.19)	0.961	0.551 – 1.676	0.8872

**Table 9:** Matched Post-Operative Complications 90 Days after Total Hip Arthroplasty (THA) SSI: surgical site infection; DVT: deep venous thrombosis; PE: pulmonary embolism

	White	Black	Comparison	CI	P Value	White	Asian	Comparison	CI	P Value	White	Hispanic	Comparison	CI	P Value
Dislocation n (%)	102 (2.12)	77 (1.60)	0.751	0.557 - 1.012	0.0601	-1 (NA)	-1 (NA)	NA	NA	NA	-1 (NA)	-1 (NA)	NA	NA	NA
SSI n (%)	139 (2.88)	127 (2.63)	0.911	0.714 - 1.163	0.4557	-1 (NA)	-1 (NA)	NA	NA	NA	13 (3.10)	-1 (NA)	NA	NA	NA
DVT n (%)	12 (0.25)	12 (0.25)	1.000	0.449 - 2.228	1.0000	-1 (NA)	-1 (NA)	NA	NA	NA	-1 (NA)	-1 (NA)	NA	NA	NA
Wound Disruption n (%)	49 (1.02)	42 (0.87)	0.856	0.566 - 1.295	0.4614	-1 (NA)	-1 (NA)	NA	NA	NA	-1 (NA)	-1 (NA)	NA	NA	NA
Hematoma n (%)	82 (1.70)	81 (1.68)	0.988	0.725 - 1.346	0.937	-1 (NA)	-1 (NA)	NA	NA	NA	-1 (NA)	-1 (NA)	NA	NA	NA
PE n (%)	42 (0.87)	43 (0.89)	1.024	0.668 - 1.570	0.9132	-1 (NA)	-1 (NA)	NA	NA	NA	-1 (NA)	-1 (NA)	NA	NA	NA
Transfusion n (%)	112 (2.32)	149 (3.09)	1.341	1.046 - 1.719	0.0207	-1 (NA)	-1 (NA)	NA	NA	NA	-1 (NA)	11 (2.6)	1.499	0.821 - 2.735	0.1872
Readmission n (%)	564 (11.8)	562 (11.8)	0.996	0.880 – 1.128	0.9494	33 (12.3)	22 (8.21)	0.637	0.361 – 1.242	0.1197	46 (11.0)	45 (11.0)	0.976	0.631 – 1.508	0.9116

**Table 10:** Matched Post-Operative Complications One Year after Total Hip Arthroplasty (THA) SSI: surgical site infection; DVT: deep venous thrombosis; PE: pulmonary embolism

	White	Black	Comparison	CI	P Value	White	Asian	Comparison	CI	P Value	White	Hispanic	Comparison	CI	P Value
Dislocation n (%)	147 (3.05)	103 (2.14)	0.694	0.538 - 0.896	0.005	-1 (NA)	-1 (NA)	NA	NA	NA	-1 (NA)	-1 (NA)	NA	NA	NA
SSI n (%)	230 (4.77)	184 (3.82)	0.792	0.650 - 0.966	0.0211	-1 (NA)	-1 (NA)	NA	NA	NA	26 (6.19)	16 (3.81)	0.782	0.392 - 1.560	0.4847
DVT n (%)	51 (1.06)	70 (1.45)	1.378	0.959 - 1.981	0.083	-1 (NA)	-1 (NA)	NA	NA	NA	-1 (NA)	-1 (NA)	NA	NA	NA
Wound Disruption n (%)	76 (1.58)	60 (1.24)	0.787	0.560 - 1.106	0.168	-1 (NA)	-1 (NA)	NA	NA	NA	-1 (NA)	-1 (NA)	NA	NA	NA
Hematoma n (%)	115 (2.38)	109 (2.26)	0.947	0.726 - 1.234	0.685	-1 (NA)	-1 (NA)	NA	NA	NA	-1 (NA)	-1 (NA)	NA	NA	NA
PE n (%)	53 (1.10)	58 (1.20)	1.096	0.753 - 1.593	0.6332	-1 (NA)	-1 (NA)	NA	NA	NA	-1 (NA)	-1 (NA)	NA	NA	NA
Transfusion n (%)	203 (4.21)	297 (6.16)	1.493	1.243 - 1.794	< 0.0001	-1 (NA)	-1 (NA)	NA	NA	NA	13 (3.10)	22 (5.24)	1.731	0.860 - 3.483	0.1243
Readmission n (%)	1502 (31.4)	1534 (32.1)	1.031	0.946 – 1.242	0.482	65 (24.3)	67 (25.0)	1.041	0.703 – 1.542	0.8411	118 (28.9)	126 (30.0)	1.097	0.8142 – 1.478	0.5432

Moreover, Black patients had a significantly lower rate of THA revision at two years (Table 11).

**Table 11:** Matched Total Hip Arthroplasty (THA) Revision Rates at Two Years

	White	Black	Odds Ratio	CI	P Value	White	Asian	Odds Ratio	CI	P Value	White	Hispanic	Odds Ratio	CI	P Value
Revisions n (%)	143 (2.97)	105 (2.18)	0.728	0.564 - 0.940	0.0149	-1 (NA)	-1 (NA)	NA	NA	NA	13 (3.10)	-1 (NA)	NA	NA	NA

Even after matching, all minority patients had significantly higher costs that became relatively higher across each time point, going from 5% at thirty days for Black patients to as high as 65% at one year for Hispanic patients (Table 12).

**Table 12:** Matched Total Costs after Total Hip Arthroplasty (in USD)

	White	Black	Comparison	CI	P Value	White	Asian	Comparison	CI	P Value	White	Hispanic	Comparison	CI	P Value
30 days avg (SD)	69761.61 (+/-48738.20)	73311.20 (+/-57056.94)	1.051	1.021 - 1.081	0.001	63803 (+/-43356.60)	93974.40 (+/- 68114.61)	1.473	1.321 - 1.625	< 0.0001	67803.77 (+/-41713.41)	96463.89 (+/-67195.27)	1.423	1.311 - 1.534	< 0.0001
90 days avg (SD)	79335.92 (+/-68313.44)	85741.72 (+/-81691.62)	1.081	1.043 - 1.119	< 0.0001	68808.88 (+/-48182.05)	104787.58 (+/- 87121.02)	1.523	1.349 - 1.696	< 0.0001	73425.92 (+/-49986.84)	111558.13 (+/-97736.22)	1.519	1.376 - 1.663	< 0.0001
1 year avg (SD)	130238.63 (+/-172829.00)	161899.74 (+/-277813.50)	1.243	1.172 - 1.314	< 0.0001	99315.56 (+/-119988.00)	172509.12 (+/-276239.60)	1.737	1.373 - 2.101	0.0001	126097.04 (+/-153972.50)	208616.11 (+/-371026.20)	1.654	1.349 - 1.960	< 0.0001

## Discussion

Using the PearlDiver database to create large cohorts of patients undergoing THA, we were able to investigate how race correlates with a set of standardized complications, revision rates, and total costs. At baseline, we observed that fewer minority patients had undergone hip replacement than White patients, similar to the previously noted phenomenon. Furthermore, we found that Black and Hispanic patients had overall poorer health compared to White patients, as indicated by the increased CCI, ECI, and LOS values and higher rates of obesity, diabetes, and tobacco use comorbidities at baseline. Previous studies have recorded similar findings [9,10], however, Elsharydah et al. [15] found Black patients to have a lower CCI score compared to White patients. We also found Asian patients to have lower CCI and ECI scores, as well as a lower prevalence of obesity and tobacco use, but an increased prevalence of diabetes. Other studies have reported the need to modify comorbidity indices to represent Asian patients more accurately [16] - how the BMI cutoff for obesity at 30kg/m^2^ can hide increased body fat percentage in Asians [17], and that the exclusion of non-English speaking Asians and Hispanics can reduce measured smoking prevalence [18,19].

Across the time points, Black patients had significantly higher rates of hematoma, transfusion, and readmission. Previous studies found similar results that Black patients had higher rates of transfusion and readmission [5,20,21]. Furthermore, Black patients had significantly higher rates of hematoma, but there was no indication in the literature that Black patients have higher rates of hematoma following THA. Therefore, more expansive research must be done into why Black patients could have higher hematoma rates than White patients. We believe this is necessary to better understand the relationship between ethnicity and hematoma formation. There were fewer patients labeled Asian or Hispanic in the PearlDiver Database which made comparisons challenging. Nonetheless, we found Hispanic patients had higher rates of transfusion at one year, similar to results found in a study by Haque et al. [22].

The matching process created a White group normalized to the characteristics of the corresponding minority patient group, meaning that the lower age and increased presence of comorbidities in minority patient groups compared to White patients at baseline was made negligible. After matching, Black patients still had significantly higher rates of transfusion at ninety days and one year, but lower rates of dislocation and SSI at one year. The rate of transfusions could be increased in Black patients due to the higher prevalence of anemia in that demographic [23]. Asian and Hispanic patients did not have any significant complications across all time points.

Interestingly, we found that the odds of undergoing revision hip surgery were similar between racial groups before matching, but it had decreased for Black patients after matching. A study by Klemt et al. [6] found a similar result: Black patients had lower utilization of revision hip replacement surgery than White patients. This is concerning as Black patients were found (in our study) to have higher rates of risk factors for THA revision, such as obesity and diabetes [24]. This suggests that there may be a racial barrier preventing Black patients from undergoing hip replacement revision surgery even when it would be beneficial for the patient.

All minority patients experienced progressively higher total costs following hip replacement both at baseline and after matching. Furthermore, Black patients’ increased rates of complications likely compound over time leading to continuously higher costs of care. Previous studies also found that minority patients had increased hospitalization, emergency room, and end-of-life costs than White patients [25]. However, Asian and Hispanic patients were not found to have higher rates of complications but still had significantly higher costs, which could be related to the increased LOS both groups experienced. This longer LOS could be a result of implicit bias as all minority patients had relatively increased LOS compared to White patients despite comorbidity matching. A similar outcome was observed by Ghosh et al. [26].

This study highlights the possibility of health disparity in Black patients who are offered primary THA. The 2020 United States Census Bureau Population Estimate reported that Caucasians represented most of the population at 61.9% and the African American Population at 12.4% was substantially less [27]. As a result, there are roughly five White patients for each Black patient in the general population. The contrast between the numbers of Black patients who underwent primary THA at 6% compared to White patients at 94% is revealing and Black patients were substantially underrepresented compared to the general population. This discrepancy is even more apparent by the comparison of Asian and Hispanic patients who are 4.8% and 18.7%, but in our study were only 0.3% and 0.5%, respectively. Furthermore, a study by Okike et al. [28] reported no substantial difference in complication rates when enrolled in a universally insured, integrated healthcare system, similar to our findings after the matching process. This suggests that many racial differences in outcomes could be ameliorated if patients had more standardized access to care, as opposed to inherent racial differences being the sole factor. Although a comparison of racial differences in patients who undergo primary THA was not the subject of this study, this disparity highlights an area that merits further research.

We recognize there are several limitations to this study. As a retrospective study with data gathered from various locations and types of practice, there were likely differences in surgical technique, equipment, and postoperative protocols. Furthermore, any procedures that were done before the index hip replacement could not be determined. Also, matching only diabetes, obesity, and tobacco use still allows for a myriad of confounding variables, such as SES, insurance type, or proximity to healthcare institutions [29], to potentially bias our results. Retrospective studies rely on the accuracy of data that was previously entered. As a result, any discrepancies in data entry due to non-standardized or subjective criteria could have affected our results. For example, many patients were categorized as “unknown” or “other” when creating the racial groups to be examined, thereby considerably limiting the number of minority patients, comprising less than 7% of our study group and decreasing our level of comparison between racial groups. Moreover, the PearlDiver Database records data based on CPT and ICD-9 codes, potentially limiting the data retrieval. Finally, the nonspecific output of -1 when values for complications were below 11 could greatly skew the resulting odds ratio and p value calculations. Nonetheless, this study benefits from a high predictive power because of the large number of patients within the PearlDiver Database. In addition, all complications and costs that were examined were decided upon before data collection began, thereby strengthening the credibility of our results. Finally, none of the authors of this study conducted the patient matching nor performed the surgeries, thereby further limiting the amount of selection bias and strengthening the credibility of our results.

## Conclusions

Our results showed significant differences in outcomes following THA based on race, with Black patients having more complications before propensity matching to their White counterparts, but more equal complications after matching. Black patients had no significant differences in revisions before matching but had decreased revision rates after matching. The total costs, however, were higher for all minority patients both before and after matching. These results will allow surgeons to better identify patients at higher risks for specific complications and enable support practices to be established to increase the utilization of THA by minority patients. By doing so, we can work to ensure patients from all racial and ethnic backgrounds do not experience health disparities but instead receive equitable healthcare.
